# An efficient point cloud semantic segmentation network with multiscale super-patch transformer

**DOI:** 10.1038/s41598-024-63451-8

**Published:** 2024-06-25

**Authors:** Yongwei Miao, Yuliang Sun, Yimin Zhang, Jinrong Wang, Xudong Zhang

**Affiliations:** 1 School of Information Science and Technology, Hangzhou Normal University, Hangzhou, 311121 China; 2 School of Information Science and Technology, Zhejiang Shuren University, Hangzhou, 310015 China; 3 School of Computer Science and Technology, Zhejiang Sci-Tech University, Hangzhou, 310018 China

**Keywords:** Computer science, Mechanical engineering

## Abstract

Efficient semantic segmentation of large-scale point cloud scenes is a fundamental and essential task for perception or understanding the surrounding 3d environments. However, due to the vast amount of point cloud data, it is always a challenging to train deep neural networks efficiently and also difficult to establish a unified model to represent different shapes effectively due to their variety and occlusions of scene objects. Taking scene super-patch as data representation and guided by its contextual information, we propose a novel multiscale super-patch transformer network (MSSPTNet) for point cloud segmentation, which consists of a multiscale super-patch local aggregation (MSSPLA) module and a super-patch transformer (SPT) module. Given large-scale point cloud data as input, a dynamic region-growing algorithm is first adopted to extract scene super-patches from the sampling points with consistent geometric features. Then, the MSSPLA module aggregates local features and their contextual information of adjacent super-patches at different scales. Owing to the self-attention mechanism, the SPT module exploits the similarity among scene super-patches in high-level feature space. By combining these two modules, our MSSPTNet can effectively learn both local and global features from the input point clouds. Finally, the interpolating upsampling and multi-layer perceptrons are exploited to generate semantic labels for the original point cloud data. Experimental results on the public S3DIS dataset demonstrate its efficiency of the proposed network for segmenting large-scale point cloud scenes, especially for those indoor scenes with a large number of repetitive structures, i.e., the network training of our MSSPTNet is much faster than other segmentation networks by a factor of tens to hundreds.

## Introduction

In the literature of 3d computer vision and visual intelligence, scene semantic segmentation is a fundamental task for understanding 3d indoor environments^1,2^. Efficient segmentation of indoor point cloud scenes, i.e. assigning a semantic label to each discrete sampling point of a scene element (such as wall, floor, ceiling, and clutter), always plays an important role in many applications of computer vision or visual robots, such as indoor navigation^3^, autonomous driving^4^, robot perception^5^, and augmented reality^6^ etc. Although deep neural networks have achieved significant breakthroughs in 2d computer vision^7^, their performance on the task of 3d point cloud semantic segmentation is still limited due to its large-scale data and non-uniform or sparse distribution of the unorganized point clouds^8,9^. The key issue of large-scale scene understanding is effectively excavating the local geometric features and context information with efficient data processing. The large-scale of point cloud data for complex 3d scenes makes it difficult for feature learning and feature extraction. The objectives of our work are to achieve efficient large-scale point cloud data processing, effective extraction of local geometric features, and effective exploration of global contextual information.

**Figure 1:**
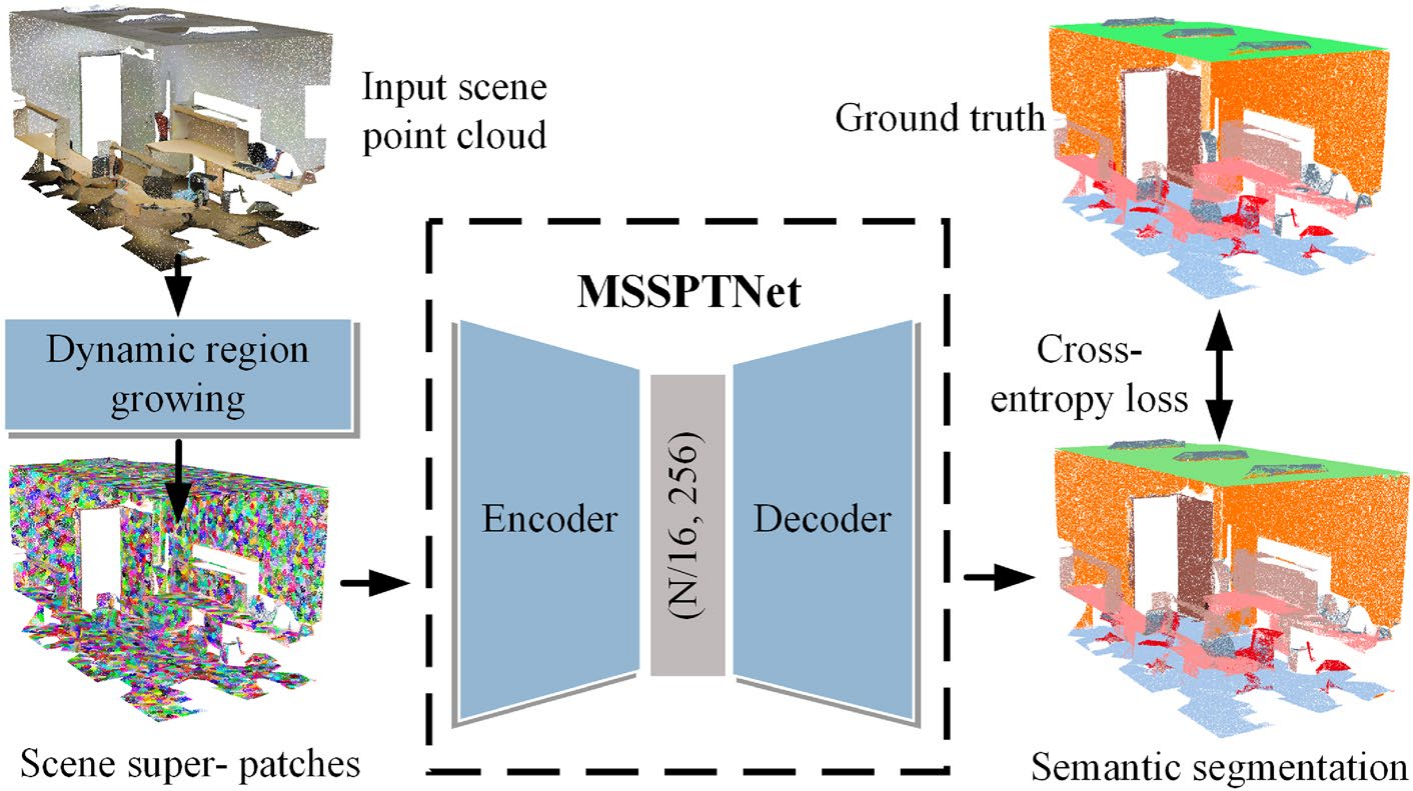
The overall pipeline of our proposed segmentation method. Scene super-patches are extracted from the input large-scale point clouds via the dynamic region growing algorithm^12^ and fed into our MSSPTNet, producing the final result of 3d semantic segmentation.

The overall structures of indoor scenes always have typical planar patches, such as walls, ceilings, floors, doors, and windows etc^10^. Most indoor furniture objects (such as tables, chairs, bookshelves, sofas) can also be represented by the combinations of multiple geometric super-patches. Here, we exploit the scene super-patches as data representation to overcome the time-consuming and memory demanding of network training for effective scene segmentation. Furthermore, to effectively segment those complex and diverse objects from indoor scenes, we design a super-patch based transformer, which will apply the self-attention mechanism introduced in the Transformer^11^ to calculate the geometric similarity between scene super-patches and thus effectively cluster the scene super-patches to generate final semantic segmentation. To effectively extract local geometric features, we design the MSSPLA module that can extract multiscale hierarchical information. To further exploit global features, we design the SPT module to explore contextual information in latent space using the self-attention mechanism. Owing to the super-patch based data representation and super-patch based transformer structure, we present a multiscale super-patch transformer network (MSSPTNet), which is context-aware and suitable for semantic segmentation of large-scale indoor scenes. The overall pipeline of our proposed segmentation method is shown in Fig. 1.

The main contributions of this work can be summarized as follows.

- Due to super-patch representation for large-scale point clouds, a context-aware transformer network MSSPTNet is presented to effectively overcome the shortcomings of inefficient and time-consuming network training for 3d semantic segmentation.
- A multiscale super-patch local aggregation (MSSPLA) module is introduced to extract and aggregate the multiscale features and context information of scene super-patches.
- A super-patch transformer (SPT) module based on self-attention is given for effectively learning the feature similarities between super-patches and also improving the performance of scene segmentation from the perspective of geometric semantics.

The rest of this paper is organized as follows. In the “Related work”, we briefly overview some related works of point cloud segmentation. The technical details of our presented point cloud segmentation network MSSPTNet are given in the “Method”, including the overall framework and key modules. Experimental results and ablation studies are given in the “Experiments”. Section “Conclusion” summarizes the research content.

## Related work

Here, we briefly review point cloud segmentation approaches which can be categorized into projection-based, discretization-based, and point-based methods.

### Projection-based and discretization-based methods

The projection-based methods firstly project 3d point clouds into 2d images and then perform the semantic segmentation task on the projected images through 2d convolution operation. The final segmentation labels are created by re-projecting the predicted image labels. Lawin et al.^13^ projected the scene point cloud data from different views to obtain synthetic images and thus applied a 2d convolution neural network (CNN) to predict the score of each pixel on the projection plane. The point labels are finally achieved by fusing the re-projection scores on different views. Similarly, Boulch et al.^14^ employed multiple cameras with different views to obtain a color map and a depth map from point clouds. They also adopted a 2d segmentation network to label each pixel and assigned labels to each sampling point by voting pixel labels. The performance of multi-view projection based methods depends on the selection of views and is sensitive to object occlusions. Tatarchenko et al.^15^ designed a network based on tangent convolutions that project surface geometry on a tangent plane. Unfortunately, the projection operation will inevitably lead to information loss and thus affect the final segmentation results. Recently, Flattening-Net^16^ succeeded in preserving geometric and topological information when converting point clouds into 2D representations. PointMCD^17^ and PointVST^18^ employed visual knowledge transferred from images to enhance point-wise embeddings.

Discretization-based methods always convert the raw point cloud data into structured and organized data form, such as voxels or lattices. Huang et al.^19^ divided the input point clouds into a set of occupied voxels, and then fed these intermediate data into 3d CNN for voxel-level segmentation and finally marked all sampling points in a common voxel using the same semantic label. Tchapmi et al.^20^ proposed a SegCloud network to achieve global consistency. This method first applied 3D-FCNN^21^ network and mapped the coarse voxels to the original point cloud through a trilinear interpolation scheme. Then, the spatial consistency of each sampling point was optimized using fully-connected conditional random field (CRF). Su et al.^22^ presented a tetrahedral lattice network SPLATNet, which interpolated 3d point clouds into a sparse lattice and thus applied convolutional operations on these lattice through bilateral convolution layers to obtain semantic segmentation. The LatticeNet presented by Rosu et al.^23^ embedded the local geometry of raw point clouds into a sparse permutohedral lattice, which can adopt for fats convolutions and thus project these lattice features back onto the point cloud data for generation of semantic labels. Lin et al.^24^ performed local flattening by mapping point clouds into 2D grids. RegGeoNet^25^ parameterized point clouds into regular 2D lattice grids for more efficient processing. These methods commonly introduce discrete artifacts and thus may lead to information loss. In general speaking, high-resolution point cloud scenes may cause high memory and computation costs, while low resolution will lead to detail loss during 3d semantic segmentation. The existing methods that transform point cloud data based on projection and discretization are challenging to avoid information loss or high computational consumption.

### Point-based segmentation methods

Recently, with the introduction of PointNet^8^ for the task of point cloud classification and segmentation, it is possible to consume discrete point cloud data as input directly. This pioneering network can learn its pointwise features using MLP layers and also extract its global latent feature uisng a max-pooling operation, which can overcome the issues of displacement invariance and rotation invariance. Subsequently, PointNet++^9^ improved PointNet^8^ by using a multiscale downsampling structure to expand the receptive field between sampling points.

To extract the potential geometric structures of point clouds for the task of semantic segmentation, Zhao et al.^26^ introduced PointWeb which can explore the relationships between sampling points through an adaptive feature adjustment module. Wang et al.^27^ employed the dynamic edge convolution for feature aggregation to learn relationships between neighboring points. However, due to its over-reliance on the transpose network, the size of their proposed DGCNN and network parameters are significantly increased, which leads to many difficulties for large-scale network training. To deal with large-scale point clouds, SPGraph^28^ represented the input data as a set of interconnected simple shapes and super-points, and exploited an attributed directed graph to extract the context information of point cloud data. However, this network is relatively complex and also time-consuming. Owing to the dilated K-nearest neighbor (DKNN) operation, Guo et al.^29^ presented a dilated multiscale fusion network for the analysis of point cloud data, especially for the task of point cloud classification and segmentation. Since the on-surface supervoxel provides a compact representation of 3d surfaces and also brings efficient connectivity structure via supervoxel clustering, Huang et al.^30^ explored the convolution operation directly on supervoxels and thus fused the multi-view 2d features and 3d features projected on these supervoxels for 2d–3d joint learning during 3d semantic prediction. To alleviate the computational costs of network training, RandLA-Net^31^ adopted a random sampling scheme to realize point cloud downsampling operation for point cloud data. This method employed a feature aggregation module to preserve geometric details and also expand the receptive field between sampling points. Although the random sampling strategy can lead to lower memory and computational cost, it may lose the intrinsic features of 3d scenes, which will cause the defects or inaccuracy for scene semantic segmentation. To improve the efficiency of large-scale point cloud learning, Part et al.^32^ designed an accelerated version of Point Transformer.

In this work, taking scene super-patches as data representation, we propose a transformer-based framework for point cloud segmentation. Compared with multi-view images or voxel representation, our method can significantly reduce the training memory and computational load. To deal with large-scale point clouds, most existing point-based approaches have limited receptive fields and are incapable of extracting local context information. We exploit scene super-patches with consistent geometry information instead of discrete point clouds to overcome these challenges. However, the super-patch representation may lose local geometric details, especially for 3d complex shapes. To counter this potential drawback, we employ a transformer-based framework combined with a multiscale local aggregation module for improving the ability of robust feature learning whilst considering the time efficiency.

## Method

Owing to scene super-patches representation and guided by their context features, in this paper we present a novel semantic segmentation framework MSSPTNet for large-scale indoor point clouds. Our method first extracts geometrically consistent patches from indoor scenes using a region-growing algorithm and calculates the geometric features of each super-patch. Then the proposed network consumes super-patches with geometric features and outputs the semantic segmentation results of 3d point cloud scenes.

**Figure 2:**
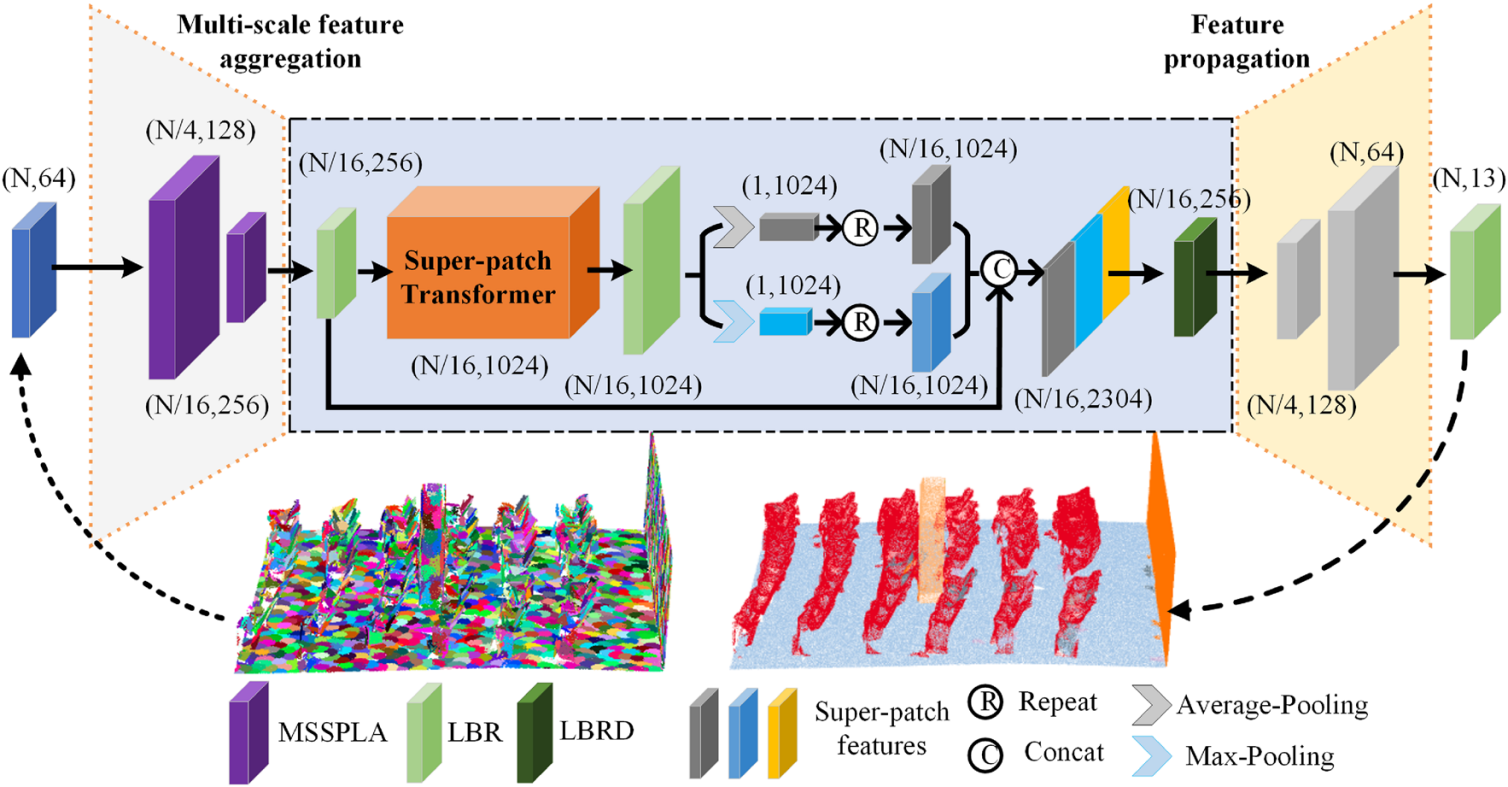
The architecture of MSSPTNet for large-scale point cloud semantic segmentation. LBR combines Linear, BatchNorm, and ReLU, and LBRD means LBR followed by a Dropout.

Figure 2 shows the architecture of our MSSPTNet which adopts an encoder-decoder structure. In the encoder, we design the MSSPLA module, which is composed of a multiscale hierarchical structure, enlarging the perception field. The super-patch local aggregation block inside the MSSPLA module is used to extract local features. Patches that are far apart in 3D space may also have the same semantic information. Therefore, global features are important. The SPT module is designed to explore global contextual information in high-level semantic latent space using the self-attention mechanism. The similarity between scene super-patches after downsampling is further studied. The decoder employs linear interpolation to restore the downsampled scene super-patch to the original scene resolution. Finally, MSSPTNet can assign semantic labels to each super-patch and outputs the segmentation results.

### Scene super-patch representation

Here, scene super-patches are exploited as the data representation of scene point clouds, which can overcome the difficulty of point-based networks for directly training large-scale data^33^. The main reasons are as follows. Firstly, the sampling points inside a super-patch are geometrically consistent and can be considered as one shape element. Secondly, the number of super-patches in the whole scene is much smaller than that of discrete sampling points, so taking super-patches as input of the network can significantly release time-consuming network training burdens. Moreover, since scene super-patches usually have better geometric representation than discrete sampling points, scene semantic segmentation can be effectively achieved by learning the context information between scene super-patches.

#### Scene super-patch generation

It is observed that man-made objects in indoor scenes are commonly constructed in a highly structured style. Inspired by Mattausch et al.^12^, we implement a clustering approach for the super-patches extraction of large-scale point cloud scenes, that is, a region-growing strategy. The idea of the region growing strategy is to first rank the input point cloud according to its curvature, and select the sample point with the highest curvature as the seed sample point ***s***. Based on the selected seed point, for the nearest neighbor point ***p*** outside the super-patch $\Pi _i$, we can check the following conditions,

1 $$\begin{aligned} {{\varvec{n}}}_{{\varvec{p}}}\cdot {{\varvec{n}}}_{{\varvec{s}}}>t_1 \end{aligned}$$

2 $$\begin{aligned} ({{\varvec{p}}}-{{\varvec{s}}})\cdot {{\varvec{n}}}_{{\varvec{s}}}<t_2 \end{aligned}$$

3 $$\begin{aligned} ({{\varvec{p}}}-{{\varvec{q}}})\cdot {{\varvec{n}}}_{{\varvec{q}}}<t_3 \end{aligned}$$

4 $$\begin{aligned} \#(\Pi _i)<t_4 \end{aligned}$$

If the conditions are satisfied, the neighbor point ***p*** will thus be added to the super-patch $\Pi _i$. Here, ***q*** means the last added sampling point inside the super-patch $\Pi _i$, and ***n*** is the normal vector of the sampling point. The conditions (Eqs. 1–3) specify the constraint that sampling point ***p*** should be close to the super-patch plane with similar normal of the seed point. When there are no sampling points that meet the above requirements or the sampling point size reaches the threshold $t_4$, a new super-patch starts growing. In practice, we employ the absolute value of vector dot products to assess vector similarity and distance. These iterations will end until all the sampling points are traversed.

#### Feature descriptors of scene super-patches

To compute the feature descriptors, the dominant axes of each super-patch can firstly be determined by PCA analysis^34^. For each projected fitting rectangle, the feature descriptors will consist of patch centroid, PCA normal, color, and fill ratio of convex hull area to area (see Table 1). These features will help the network to learn the semantic relationships between scene super-patches and always achieve accurate segmentation.

**Table 1:** Feature descriptors of the extracted super-patch.

Features	Descriptions
${\textbf {P}}_p$	Patch centroid
${\textbf {P}}_n$	PCA normal vector
${\textbf {P}}_c$	Colour
${\textbf {P}}_h$	Height
${\textbf {P}}_r$	Aspect ratio
${\textbf {P}}_a$	Area
${\textbf {P}}_f$	Area fill ratio

### Multiscale feature extraction of scene super-patch and feature aggregation

For large-scale point cloud semantic segmentation, the context information is always difficult to extract only at a single scale. Inspired by PointNet++^9^, a multiscale architecture is designed for feature extraction and aggregation in our segmentation network. We map the feature descriptors of input scene super-patches to high-dimensional space and then extract features at different scales using a two-layer MSSPLA module consisting of a Super-Patch Local Aggregation (SPLA) block.

#### Multiscale feature extraction of scene super-patch

**Figure 3:**
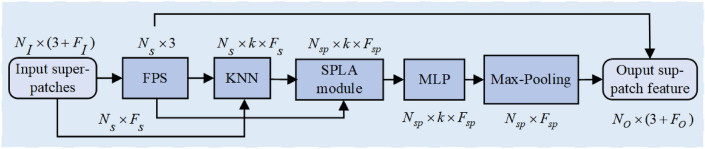
MSSPLA module for multiscale super-patch feature extraction.

Local features of scene point clouds always play a crucial role in the task of semantic segmentation. To extract the abundant features in super-patch data representation, we design and employ a MSSPLA module to extract super-patch features at different scales. As shown in Fig. 3, scene super-patches $\Pi _I\in R^{N_I\times (3+F_I)}$ generated from the original point cloud are employed as the input of the MSSPLA module. Here, $N_I$ is the total number of scene super-patches, and each super-patch contains $F_I$ dimensional feature and 3d centroid coordinates. Firstly, we employ the farthest point sampling algorithm (FPS)^35^ to obtain the downsampled super-patches $\Pi _s\in R^{N_s\times (3+F_s)}$. This step picks a seed super-patch from the input super-patches and iteratively selects $N_s$ super-patches with the farthest Euclidean distances between their centroids. The number of super-patches is reduced from $N_I$ to $N_s$ with doubled feature dimension. Furthermore, to obtain the context information for downsampled super-patches $\Pi _s$, we employ the KNN algorithm^36^ to obtain the nearest neighbor super-patches from the input scene super-patch. This step can find its k-nearest super-patches $\Pi _s^k\in R^{N_s\times F_s}$ from $\Pi _i$, and each super-patch contains $F_s$ dimensional feature. Then, through the local feature aggregation operation, we can employ the SPLA block to obtain $N_{sp}$ super-patches $\Pi _{sp}^k\in R^{N_{sp}\times F_{sp}}$, where each super-patch contains $F_{sp}$ dimensional feature. The feature dimension of the super-patch is then reduced using multi-layer perceptions (MLPs), and the global features are extracted by the max-pooling operation. Finally, the output super-patch features $\Pi _{sp}^k$ are concatenated with the downsampled super-patches $\Pi _s$ to form the final scene representation $\Pi _O\in R^{N_O\times (3+F_O)}$. Here, each super-patch contains $F_O$ dimensional feature and 3d centroid coordinates.

As shown in Fig. 3, KNN means K-Nearest Neighbor algorithm, and SPLA is the scene super-patch local aggregation block. In our presented segmentation network, if the number of scene super-patch is 1024 with 64-dimension features, the number of super-patches will firstly reduce to 256 using the downsampling algorithm, and their feature dimension is increased to 128. The SPLA block is then employed to aggregate local features of scene super-patches to obtain 256-dimensional features. The total 256 super-patches with 128-dimensional features can finally be obtained through multi-layer perceptron and max-pooling operation.

#### Local feature aggregation of scene super-patches

**Figure 4:**
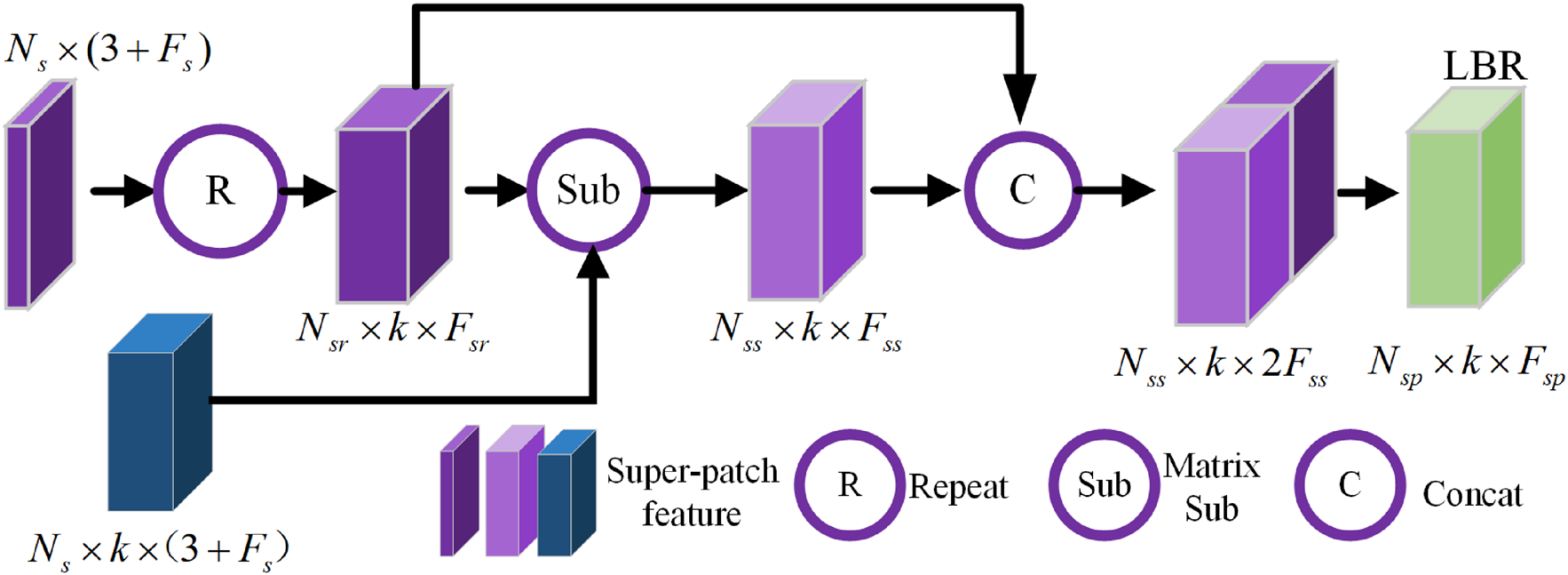
SPLA block for super-patch local feature aggregation.

To effectively capture the context information between scene super-patches, a local feature aggregation block (SPLA) is designed and employed as a component of the MSSPLA module, as shown in Fig. 4. The scene super-patches extracted from point clouds always have rich geometric features, and we can thus further aggregate context information by computing their feature difference between adjacent super-patches and central ones. The input data of SPLA contains the downsampled scene super-patches $\Pi _s\in R^{N_s\times (3+F_s)}$ and their corresponding k-nearest super-patches $\Pi _s^k\in R^{N_s\times (3+F_s)}$. To compute the feature difference of super-patches, the feature matrix $F_s^r({{\varvec{p}}})$ is obtained by firstly broadcasting the feature vectors $F({{\varvec{p}}})$ of the downsampled super-patches. Then we compute the feature difference between each super-patch and all adjacent super-patches, producing a geometric feature difference matrix $\Delta F({{\varvec{p}}})$. This matrix is thereafter concatenated with $F_s^r({{\varvec{p}}})$ and followed by the LBR layer. This procedure can be formulated as follows,

5 $$\begin{aligned} F_s^r({{\varvec{p}}})=Repeat[F({{\varvec{p}}}),k] \end{aligned}$$

6 $$\begin{aligned} \Delta F({{\varvec{p}}})=concat_{{{\varvec{q}}}\in KNN({{\varvec{p}}},\Pi _i)}[F({{\varvec{q}}})-F({{\varvec{p}}})]\end{aligned}$$

7 $$\begin{aligned} F_s^s({{\varvec{p}}})=concat[\Delta F({{\varvec{p}}}), F_s^r({{\varvec{p}}})]\end{aligned}$$

8 $$\begin{aligned} F_{sp}({{\varvec{p}}})=Relu\{BN[MLP(F_s^s({{\varvec{p}}}))]\} \end{aligned}$$

here, BN means batch normalization, and Relu is an activation function.

### Scene super-patch transformer (SPT) module

To further extract the global information of 3d scenes, we employ the Transformer structure which performs better than traditional convolution network^37,38^. As shown in Fig. 2, the purpose of embedding the Transformer module in our MSSPTNet is to map the geometric features of the scene super-patches into a higher-dimensional semantic space, where the feature similarity between scene super-patches can be effectively learned. The input of the SPT module is the super-patch features obtained by the MSSPLA module, and the global features are learned and output through four stacked attention layers. Finally, these global and local features are concatenated for subsequent semantic segmentation.

#### Super-patch transformer

**Figure 5:**
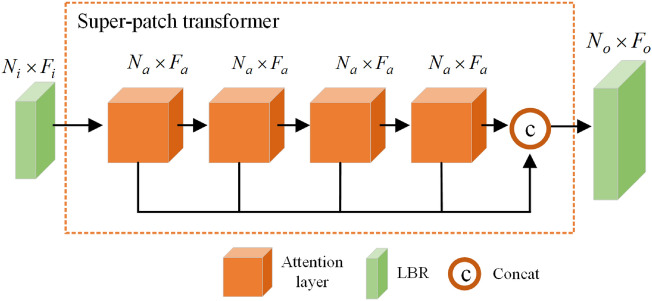
SPT module for super-patch context information extraction.

To effectively learn the feature similarity between super-patches in semantic latent space, we stack four attention layers. As shown in Fig. 5, given the input data $\Pi _I\in R^{N_I\times (3+d_I)}$ generated by the MSSPLA module, which consists of $N_I$ super-patches with $d_I$ dimensional feature and 3-dimension centroid coordinates. The super-patch feature ${{\varvec{F}}}_e\in R^{N_e\times d_e}$ is firstly obtained by the nested layers of features. Then, we can sequentially construct the high-level semantic latent space using four attention layers, where the output features of each attention layer are ${{\varvec{F}}}_a\in R^{N_a\times d_a}$. Finally, the final output features ${{\varvec{F}}}_O\in R^{N_O\times d_O}$ can be obtained by linear transformation layer, where $d_e=d_a=d_O/4$. The procedure can be formulated as follows,

9 $$\begin{aligned} {{\varvec{F}}}_1=AT^1({{\varvec{F}}}_e)\end{aligned}$$

10 $$\begin{aligned} {{\varvec{F}}}_2=AT^2({{\varvec{F}}}_1)\end{aligned}$$

11 $$\begin{aligned} {{\varvec{F}}}_3=AT^3({{\varvec{F}}}_2)\end{aligned}$$

12 $$\begin{aligned} {{\varvec{F}}}_4=AT^4({{\varvec{F}}}_3)\end{aligned}$$

13 $$\begin{aligned} {{\varvec{F}}}_O=concat({{\varvec{F}}}_1,{{\varvec{F}}}_2, {{\varvec{F}}}_3, {{\varvec{F}}}_4)\cdot {{\varvec{W}}}_o \end{aligned}$$

where $AT^i$ means the *i*-th attention layer, each attention layer has the same output dimension as that of the input, and $W_o$ represents the weights of the LBR layer.

#### Offset-attention mechanism

Self-attention mechanism can be used to compute the semantic relationships between different input data in a data sequence^11^. The key idea is to obtain the query matrix, the key matrix, and the value matrix by a linear transformation of the input feature ${{\varvec{F}}}_I$, and then to calculate their correlation between the input feature by matrix dot-product and normalization operations to obtain the attention matrix. That is, the output feature ${{\varvec{F}}}_{sa}$ from the attention layer is the weighted sum of the value matrix ***V*** and the attention weights ***A*** as follows,

14 $$\begin{aligned} {{\varvec{F}}}_{sa}={{\varvec{A}}}\cdot {{\varvec{V}}} \end{aligned}$$

Furthermore, we replace the self-attention with an offset-attention mechanism, which can enhance its processing ability of Transformer structure for point cloud data^37,38^. So, the final output feature is the combination of input feature ${{\varvec{F}}}_I$ and the LBR transformation of ${{\varvec{F}}}_I-{{\varvec{F}}}_{sa}$ as follows,

15 $$\begin{aligned} {{\varvec{F}}}_O=OA({{\varvec{F}}}_I)=Relu\{BN[MLP({{\varvec{F}}}_I-{{\varvec{F}}}_{sa})]\}+{{\varvec{F}}}_I \end{aligned}$$

The normalization of offset attention can be implemented using the softmax function and the normalization function as follows,

16 $$\begin{aligned}{}[\alpha _o]_{i,j}=softmax[(\alpha _o)_{i,j}]=exp[(\alpha _o)_{i,j}]/\sum _{k}exp[(\alpha _o)_{k,j}]\end{aligned}$$

17 $$\begin{aligned} \alpha _{i,j}=[\alpha _o]_{i,j}/\sum _{k}[\alpha _o]_{i,k} \end{aligned}$$

The introduced offset attention has the following advantages. Firstly, it will support parallel computation and can effectively capture both local and global context information of scene super-patches. Secondly, more computational power can be applied to those features with high attention, which improves the explanatory ability of the network model. Thirdly, since different super-patches will focus on different scene regions, the difference between input features and self-attention features can be obtained more effectively by offset attention, which is beneficial for the task of super-patch based semantic segmentation.

## Experiments

The proposed point cloud segmentation network has been implemented on Ubuntu 16.04 using the Pytorch framework with an Nvidia GeForce RTX 3060 graphics card. We choose the cross-entropy as the loss function and Adam as the optimizer. The network is trained for 300 epochs with a batch size of 32. The learning rate is set to 0.0005. Our method exploits the scene super-patches as data representation, which are firstly extracted from the input point cloud using a region-growing algorithm. Then, the MSSPLA module is employed to extract the super-patch features at different scales, and the SPT module is introduced to learn the high-level scene semantic features. The decoder can restore the downsampled super-patch to that of the original scene resolution, which will finally be assigned with the semantic labels.

### Datasets and data preprocessing

To verify the effectiveness and robustness of our proposed MSSPTNet, we run the performance evaluation on the S3DIS dataset^10^. The dataset contains 6 large-scale indoor scenes, including 272 rooms in total. Each room contains a 3d point cloud with the ground truth annotation, and each sampling point is labeled with a semantic label from 13 categories (ceiling, floor, wall, beam, column, window, door, table, chair, sofa, bookcase, board, clutter). Among these 3d scenes, Area 2 contains the grand theater areas with more than 10 million sampling points, each containing lots of repetitive structures. Area 5 is favorable for evaluating generality in previous studies. Here we choose these two indoor scenes as testing data and train on others.

For the input large-scale point cloud data, scene super-patches are generated through the region-growing scheme. The maximum and minimum point numbers of each super-patch are set to 128 and 30, respectively. The geometric feature descriptor of each super-patch is thus calculated. To capture the clear boundaries of different objects for efficient semantic segmentation, the input point cloud scenes are partitioned into several blocks, where the number of super-patches in each block is fixed, and one super-patch always belongs to one single block.

### Segmentation results via MSSPTNet

**Figure 6:**
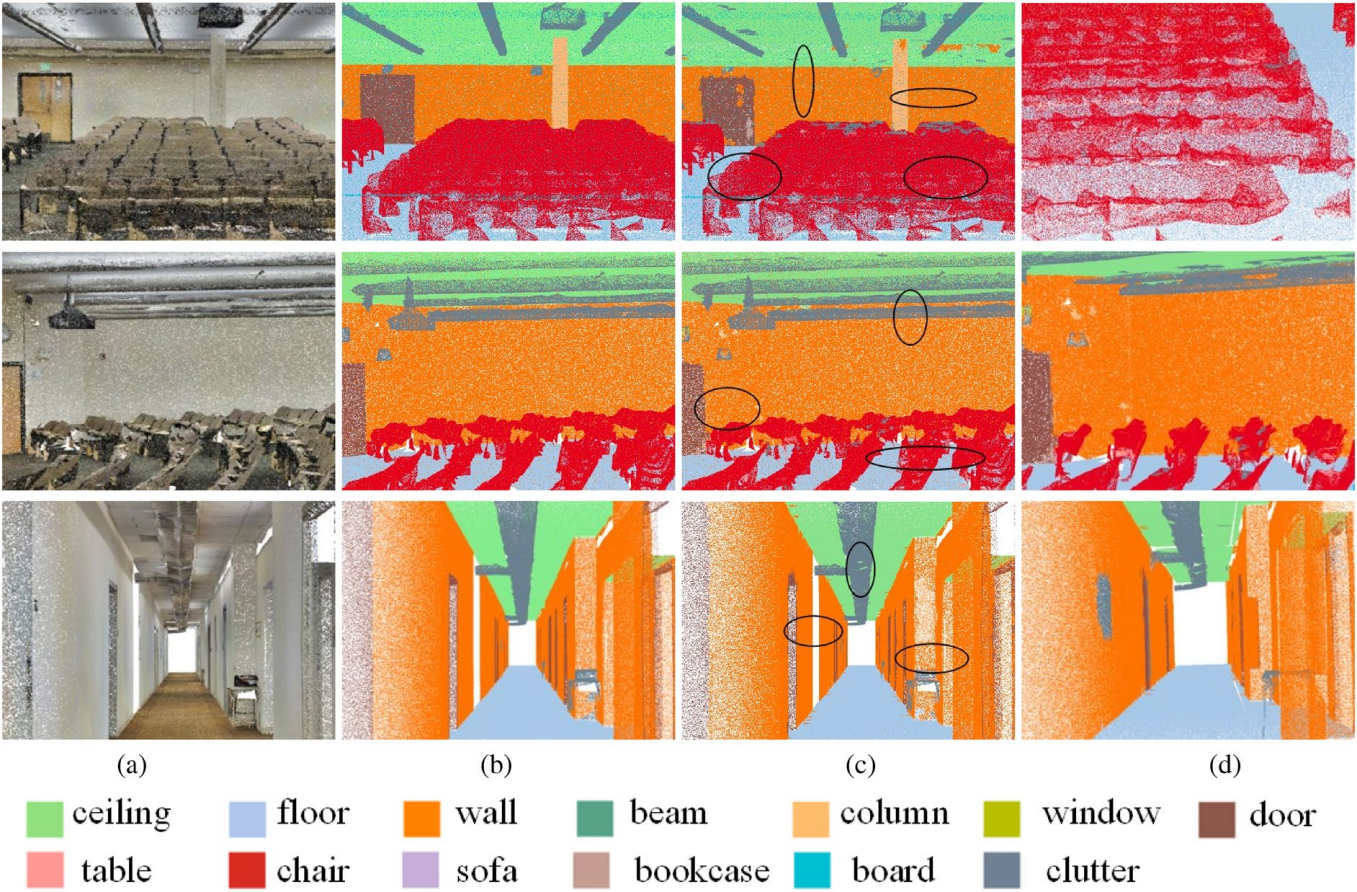
Segmentation results on theater scenes in Area 2 of S3DIS dataset. For each point cloud scene, (**a**) gives the input model and (**b**) shows the corresponding ground truth of semantic labels; (**c**) shows the segmentation results via our proposed network, and (**d**) gives their zoom-in views.

**Figure 7:**
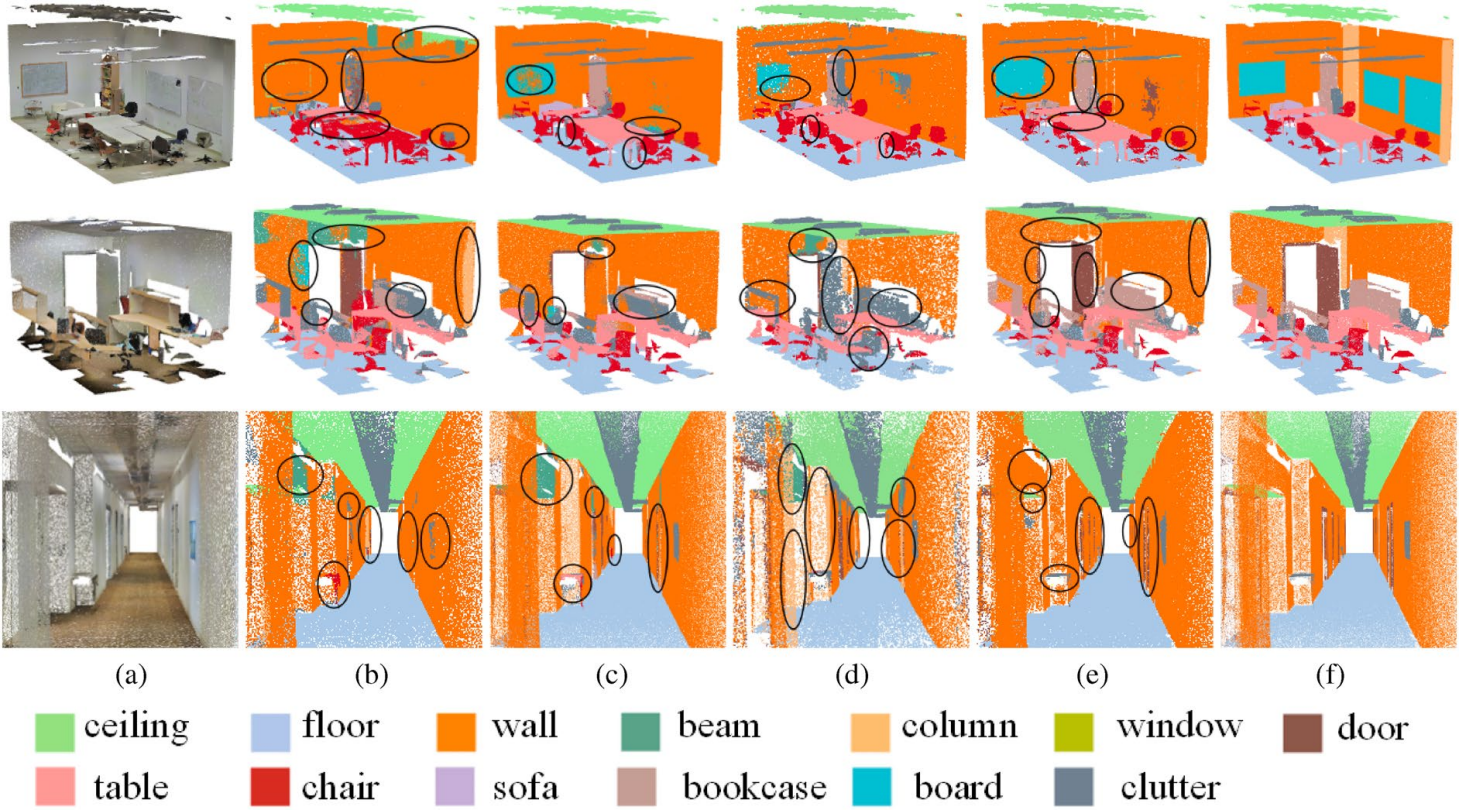
Segmentation results obtained by PointNet^8^, PointNet++^9^, DGCNN^27^ and our MSSPTNet on S3DIS dataset. For each point cloud scene, (**a**) gives the input model; (**b**) shows the segmentation results via PointNet^8^; (**c**) shows the segmentation results via PointNet++^9^; (**d**) shows the segmentation results via DGCNN^27^; (**e**) shows the segmentation results via our proposed network; (**f**) gives the corresponding ground truth of semantic labels.

The proposed network MSSPTNet has a prominent effect on the segmentation of large-scale scenes with repetitive structures. Figure 6 shows the semantic segmentation results of theater and corridor scenes in Area 2 from the S3DIS dataset^10^. For a theater scene with 10 million sampling points, our MSSPTNet can achieve efficient segmentation results with less computing costs. As shown in Fig. 6, the scene structure is completely segmented whilst the boundaries of different objects are clear (see in black ellipse). It can be seen from the zoom-in views in the 4th column of Fig. 6 that the chairs, ceilings, floors, doors, walls, and columns in the large-scale scenes can be effectively segmented. In particular, objects with repetitive structures, such as theatre chairs in row 2 of Fig. 6, can be annotated accurately. As shown in the corridor scene in row 3 of Fig. 6, most of the building elements can be accurately segmented with their structural preserved. Moreover, the wall elements can be segmented effectively even while they are disturbed by the nearby columns, beams, and clutters, thus demonstrating the resistance of our introduced network to interference. Furthermore, to verify the robustness of the proposed network, the semantic segmentation results of different scenes are also tested using different training sets as shown in Fig. 7. The experiments illustrate that our presented network is effective in segmenting large-scale point cloud scenes, especially for those scenes containing lots of repetitive structures.

### Time efficiency of MSSPTNet

The key advantage of our proposed MSSPTNet is its time efficiency due to the scene super-patches extraction and data representation for large-scale point clouds, which can vastly reduce the size of network inputs and greatly accelerates network training. Table 2 lists the time statistics of different segmentation networks. We choose Area 5 from the S3DIS dataset^10^ as the training dataset, which contains 204 training scenes in 5 areas with 195 million sampling points. The training time of different networks is computed under the same hardware environment, using the same running echos and batch size. As shown in Table 2, the training time of PointNet^8^ and DGCNN^27^ is 105 min and 175 min, respectively. Since PointNet++^9^ employs hierarchical feature extraction, its network training time increases to nearly 557 min. The network framework of SPGraph^28^ is complex, which combines graph convolution and PointNet^8^. It takes about 2471 min for network training. On the contrary, the training time of our MSSPTNet takes only 15 min, which is much faster than other networks by a factor of tens to hundreds. The inference time of our network is significantly lower than other methods.

**Table 2:** Time statistics of different networks using the same inputs.

Method	Data representation	Parameter size	Training time (min)	Inference time (min)
PointNet^8^	Sampling points	1.93M	105	33
PointNet++^9^	Sampling points	0.97M	557	185
DGCNN^27^	Sampling points	0.99M	175	45
SPGraph^28^	Super-points	0.25M	2471	864
Ours	Super-patches	5.12M	15	6

### Comparisons of different methods

To demonstrate the effectiveness of our proposed MSSPTNet, we employ the following networks for performance comparison: (1) PointNet^8^: this pioneering work combines the local features with the global features to achieve the segmentation results; (2) PointNet++^9^: this extended network adopts the encoder–decoder structure to extract multiscale features of sampling points; (3) DGCNN^27^: this network employs a dynamic graph convolution operation for local feature aggregation; (4) RandLa-Net^31^: this network tries to solve time-consuming issue in segmenting the large-scale scenes by using random sampling. We test these methods on the same dataset and hardware environments.

#### Qualitative comparisons

Figure 7 shows the semantic segmentation results of Area5 from the S3DIS dataset^10^ via different methods. The black ellipses highlight the segmentation differences via different networks. It can be seen that PointNet^8^ will fail to segment such as blackboards, tables, doors, bookshelves, etc. PointNet++^9^ and DGCNN^27^ can better segment blackboards and tables because these two methods can explore local features and the relationships between sampling points. However, these two networks still have difficulty identifying boundaries and extracting objects such as doors and bookcases. Our proposed network, which adopts scene super-patches as data representation and super-patch context information as guidance, can achieve more accurate segmentation performance. As seen from the black ellipses, our method can generate richer and clearer boundaries of objects while maintaining their detailed structures such as table legs. As shown in Fig. 7, the segmentation results of our presented network outperform that of DGCNN^27^ in terms of doors, walls, clutters, and chairs. The segmentation results of the conference room scene in Fig. 7 demonstrate that our MSSPTNet has shown better performance at extracting local features, and it can achieve better segment results for boards, bookcases, etc. Our segmentation network exploits scene super-patches as data representation and is guided by their contextual information, which can significantly improve the segmentation performance than that of PointNet^8^ and DGCNN^27^. Furthermore, due to its super-patch representation, our segmentation network has great advantages in training efforts, running 7 times or even 165 times faster than the other three networks.

**Figure 8:**
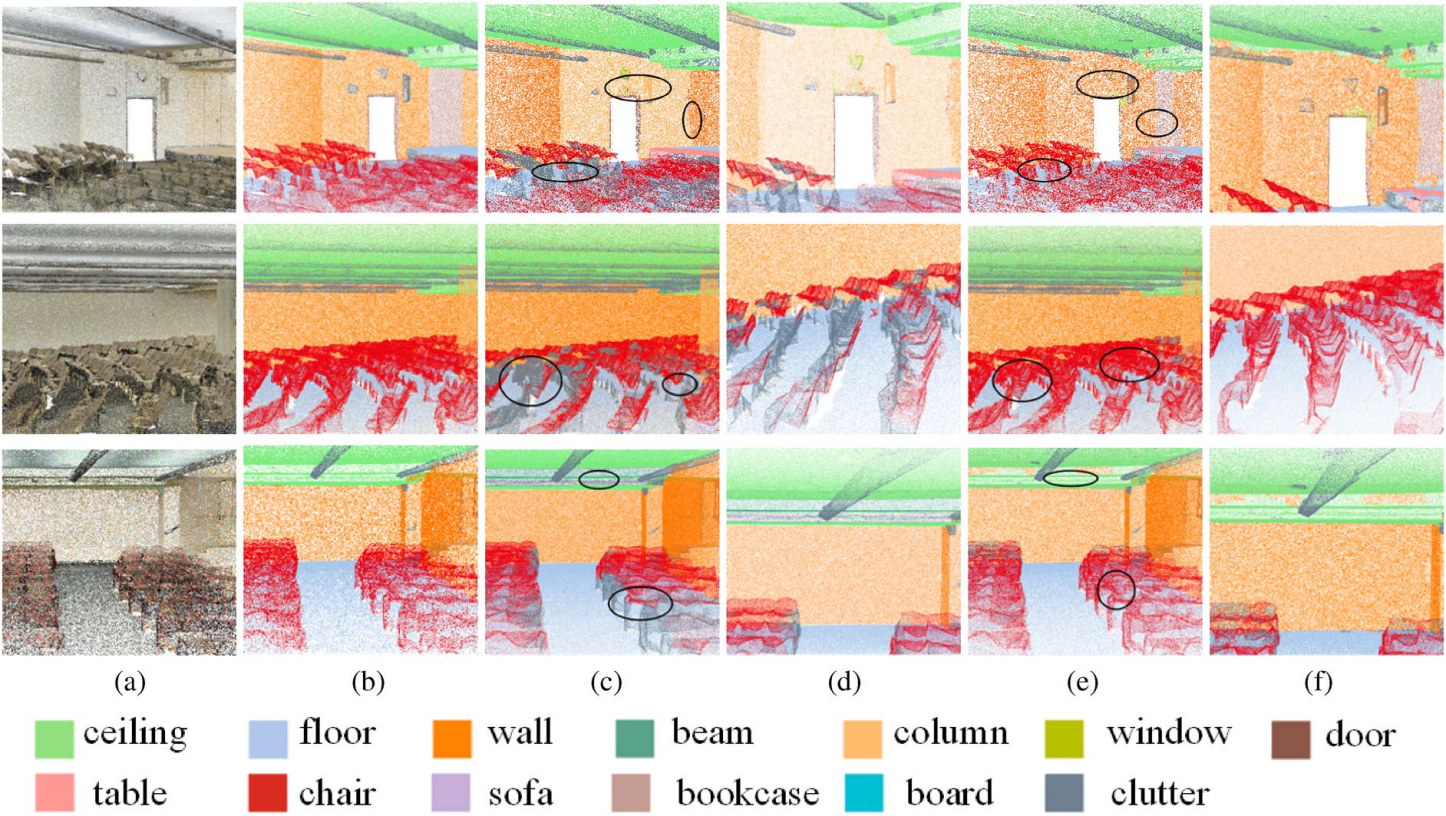
Segmentation results of Area2 from S3DIS dataset^10^ via different networks. For each point cloud scene, (**a**) gives the input point cloud scenes and the corresponding ground truth of semantic labels in (**b**); (**c**) shows the segmentation results via RandLa-Net^31^ and their zoom-in views in (**d**); (**e**) shows the segmentation results via our proposed network and their zoom-in views in (**f**).

We also compare the segmentation results with RandLA-Net^31^ in Area 2 of the S3DIS dataset^10^. As shown in Fig. 8, RandLA-Net^31^ may incorrectly classify the chairs as clutters, and also classify others as walls. Since RandLA-Net^31^ employs a random sampling strategy to reduce the size of sampling points, it is inevitable that some detailed geometric information may miss and thus brings out blurred boundaries. On the contrary, our network can generate more accurate segmentation results of those categories with sharp boundaries, such as bookcases and chairs, etc. Our super-patch representation can be beneficial to generate better performance on scene segmentation by keeping local geometry consistent. The introduced hierarchical MSSPLA and SPT module can aggregate multiscale features and exploit contextual information in the high-level semantic latent space, resulting in accurate segmentation of indoor objects.

#### Quantitative comparisons

The quantitative performance of different segmentation networks is evaluated on Area 5 from the S3DIS dataset^10^ in terms of the following metrics: overall accuracy (OA), class-wise mean of accuracy (mAcc), per-class intersection over union (IoU) and class-wise unweighted average of IoU (mIoU). For the performance evaluation listed in Table 3, the higher values indicate better segmentation results. Experiments show that the mIoU of our presented network is higher than that of PointNet^8^ and DGCNN^27^, whilst the IoU of our presented network is higher than those of the above two networks for many individual categories, such as ceiling, floor, wall, column, door, table, chair, sofa, and board. It demonstrates that our Transformer structure has better performance on feature extraction than traditional convolution networks. The EdgeConv module introduced in DGCNN^27^ can learn relationships between sampling points using offset computation. Nevertheless, it may neglect the normal vectors of neighboring points and other features. On the contrary, our presented network considers the similarity between normal vectors and other geometric features of scene super-patches, which can achieve better performance on segmenting point clouds. Our method can achieve competitive results compared to more recent point-based approaches such as PointWeb^26^, and PCT^38^. In particular, our network reaches the best and the second-best performance floor and ceiling, respectively. In comparison with SegCloud^20^, the accuracy of our presented network is higher for most categories. Their voxelization processing may lose some local geometric features, nevertheless, our super-patch representation can generate better segmentation results with much lower computation costs. Our segmentation results are also better than that of SPGraph^28^ in some categories such as ceiling, floor, beam, chair, and board. Though the recent works StratifiedPT^39^ and SPT^40^ achieved better mAcc and mIoU, our method can always produce the competitive segmentation performance of room layouts. However, the training efficiency of our presented network is about 165 times faster than SPGraph^28^, as discussed in the next section. Our proposed network can effectively maintain the overall indoor structure and achieve competitive segmentation accuracy.

**Table 3:** Quantitative performance comparisons in Area 5 of S3DIS dataset^10^.

Method	OA	mAcc	mIoU	Ceiling	Floor	Wall	Column	Window	Door	Table	Chair	Bookcase	Clutter
PointNet^8^	–	48.98	41.09	88.80	97.33	69.80	3.92	46.26	10.76	58.93	52.61	40.28	33.22
DGCNN^27^	82.20	58.26	49.60	88.13	97.41	71.40	4.88	45.50	32.29	70.99	59.11	45.33	35.43
PointWeb^26^	–	66.64	60.28	91.95	98.48	79.39	21.11	59.72	34.81	88.27	76.33	46.89	52.46
PCT^38^	–	67.65	61.33	92.54	98.42	80.62	19.37	61.64	48.00	85.20	76.58	46.22	52.29
StratifiedPT^39^	91.50	78.10	72.00	96.20	98.70	85.60	46.10	60.00	76.80	84.50	92.60	77.80	64.00
^†^SegCloud^20^	–	57.35	48.92	90.06	96.05	69.86	18.37	38.35	23.12	70.40	75.89	58.42	41.60
^‡^SPGraph^28^	86.38	66.50	58.04	89.35	96.87	78.12	42.81	48.93	61.58	84.66	75.41	52.60	52.22
^‡^SPT^40^	–	–	68.90	92.60	97.70	83.50	42.00	60.60	67.10	81.00	88.80	73.20	60.00
Ours	84.05	62.74	50.35	92.25	98.63	73.92	17.49	38.48	46.08	72.24	76.12	45.24	34.11

*$\dag$ and $\ddag$ are voxel-based and superpoint-based data presentation, respectively. Our method uses super-patch representation.

### Ablation study

#### Effect of super-patch features

To analyze the influence of scene super-patch features, we conduct an ablation study on Area 5 from the S3DIS dataset^10^ using different components of feature descriptor. We employ OA, mAcc, and mIoU as evaluation metrics. The baseline is the best segmentation result using only super-patch centroid coordinates. The normal, RGB color, and other geometric information, as in Table 4, are added in turn. This ablation study demonstrates that normal information contributes most to the segmentation task, which accounts for a 3.5 gain in mIoU. It can be seen from this ablation study that RGB color and other geometric features also play important roles in the final segmentation results.

**Table 4:** Ablation experiments on input super-patch features and network modules.

Module	Super-patch feature	OA	mAcc	mIoU
Full network	xyz	77.77	56.74	45.54
Full network	xyz + normal	83.28	60.78	49.04
Full network	xyz + normal + rgb	84.00	62.72	50.28
Full network	Full features	84.05	62.74	50.35
noSPLA	Full features	79.55	58.84	46.95
noSPT	Full features	76.65	53.64	42.15

#### Effect of network modules

To further study the influence of different components introduced in our network, we conduct an ablation study on Area 5 from the S3DIS dataset^10^. We use the full network with full super-patch features as a baseline and present the segmentation results of different choices in terms of OA, mAcc, and mIoU. noSPLA removes the SPLA block and noSPT removes the SPT module. As shown in Table 4, the SPT module greatly influences the semantic segmentation results, which accounts for reducing 8.20 mIoU performance. Moreover, if the local feature extractor SPLA block is missing, the model performance of the segmentation network will be significantly reduced by 3.40 for mIoU performance. This ablation study convincingly validates the effectiveness and benefit of our design choices.

## Conclusion

In this paper, we propose a novel context-aware super-patch transformer network MSSPTNet for large-scale point cloud segmentation. Scene super-patches with consistent geometric features are used as input, and the context information is learned through an encoder-decoder structure. The encoder can effectively gather the information of adjacent super-patch at different scales by embedding the local feature aggregation module. Furthermore, to better learn the semantic relationships between scene super-patches, the Transformer module based on a self-attention mechanism is employed. The experimental results demonstrate the efficiency of the proposed network for large-scale point cloud segmentation, especially for those indoor scenes with a large number of repetitive structures.

However, our method can not achieve the desired segmentation results in messy scenes with complex object shapes. It is difficult to distinguish those objects with similar planar shapes. Therefore, we will attempt to improve the local feature aggregator to boost the performance of large-scale point cloud segmentation. Although MSSPTNet is primarily designed for indoor scenes, in the future, we could extend the proposed method to the task of point cloud segmentation for 3d outdoor scenes.

## Data Availability

The datasets analyzed during the current study are available at S3DIS dataset [http://buildingparser.stanford.edu].
